# Flame Retardancy and Toughness of Poly(Lactic Acid)/GNR/SiAHP Composites

**DOI:** 10.3390/polym11071129

**Published:** 2019-07-03

**Authors:** Ningjing Wu, Jihang Yu, Wenchao Lang, Xiaobing Ma, Yue Yang

**Affiliations:** 1 Key Laboratory of Rubber-Plastics, Ministry of Education/Shandong Provincial Key Lab of Rubber-Plastics, Qingdao University of Science & Technology, Qingdao 266042, China; 2 School of Chemistry, Sun Yat-sen University, Guangzhou 510275, China

**Keywords:** PLA composite, flame retardancy, toughness, melt-dripping resistance, surface modification

## Abstract

A novel flame-retardant and toughened bio-based poly(lactic acid) (PLA)/glycidyl methacrylate-grafted natural rubber (GNR) composite was fabricated by sequentially dynamical vulcanizing and reactive melt-blending. The surface modification of aluminum hypophosphite (AHP) enhanced the interfacial compatibility between the modified aluminum hypophosphite by silane (SiAHP) and PLA/GNR matrix and the charring ability of the PLA/GNR/SiAHP composites to a certain extent, and the toughness and flame retardancy of the PLA/GNR/SiAHP composites were slightly higher than those of PLA/GNR/AHP composites, respectively. The notched impact strength and elongation of the PLA composite with 20 wt. %GNR and 18 wt.% SiAHP were 13.1 kJ/m^2^ and 72%, approximately 385% and 17 fold higher than those of PLA, respectively, and its limiting oxygen index increased to 26.5% and a UL-94 V-0 rating was achieved. Notedly, the very serious melt-dripping characteristics of PLA during combustion was completely suppressed. The peak heat release rate and total heat release values of the PLA/GNR/SiAHP composites dramatically reduced, and the char yield obviously increased with an increasing SiAHP content in the cone calorimeter test. The good flame retardancy of the PLA/GNR/SiAHP composites was suggested to be the result of a synergistic effect involving gaseous and condensed phase flame-retardant mechanisms. The high-performance flame-retardant PLA/GNR/SiAHP composites have great potential application as replacements for petroleum-based polymers in the automotive interior and building fields.

## 1. Introduction

Bio-based poly(lactic acid) (PLA) is the most promising replacement for traditional petroleum-based polymers because of its biodegradability, sustainability, and good mechanical strengths. However, PLA is inherently highly inflammable, with a limiting oxygen index (LOI) of only 19%, and it has a very serious melt-dripping problem. In addition, the disadvantages of brittleness and low thermal deformation temperature, extremely restrict its potential application in engineering plastics fields [1,2].

A variety of halogen-free flame retardants including inorganic phosphorous-containing flame retardants [3,4,5,6], intumescent flame retardants (IFRs) [7,8,9], zinc–aluminum-layered double hydroxide [10], and modified layered double hydroxides [11] were used to improve the flame retardancy of PLA. Aluminum hypophosphite (AHP) is an efficient flame retardant for PLA/basalt fiber (BF) composites. With the addition of 19 wt.% AHP and 1 wt.% modified carbon nanotubes (m-CNTs), the PLA/AHP/m-CNR composite achieved a V-0 rating and an LOI value of 31% [3]. In general, the addition of inorganic phosphorous-containing flame retardants, IFRs or metal hydroxides endows PLA with good flame retardancy. However, the usage of the additive flame retardant generally causes further deterioration through the brittleness of the PLA composites due to the poor compatibility between flame retardants and the PLA matrix. 

Some highly efficient organo-phosphorus-containing flame retardants, including phosphates [12], aluminum diethylphosphinate [13], phosphorus-containing diacid [14,15], 9,10-dihydro-9-oxa-10-phosphaphenanthrene-10-oxide (DOPO) derivatives [16], phosphazene-triazine compounds [17], flame-retardant-containing phosphorus–nitrogen silicon [18], and polymeric flame retardant [19], have recently been used to improve the flame retardancy of PLA. With only 3 wt.% poly(phenylphosphoryl phenylenediamine) melt-blended with PLA, a V-0 rating in the UL-94 testing was achieved and the LOI value increased to 25.5% [20]. To maintain the sustainability of bio-based polymer, the applications of some bio-based flame retardants in PLA have also received much attention [21,22,23,24]. A bio-based polyphosphonate (BPPT) based on plant-derived diphenolic acid (DPA) at 4 wt.% loading was incorporated into PLA to achieve a UL-94 V-0 rating [22]. The investigation of flame-retardant mechanisms demonstrates that the organo-phosphorus-containing flame retardants were mainly ascribed to phosphorous-containing radical inhibition. Meanwhile, some of the combustion heat of the flame-retardant PLA materials was carried away by melt-dripping during the combustion process [17,19]. Thus, the high efficiency flame-retardant PLA materials could not completely solve the melt-dripping problem. Some bio-based additives such as chitosan [4], starch [8], and cellulose nanofiber [24] have been incorporated to improve the flame retardancy and mechanical properties of PLA composites. A kind of modified cellulose nanofiber (PN-FR@CNF) with 10 wt.% loading enhanced the tensile strength of PLA by approximately 24% and achieved a V-0 rating [25]. However, these biodegradable polysaccharide fillers severely embrittled the PLA matrix, making it difficult to process [24,25].

Nowadays, super-tough PLA materials have led to considerable attention, which have been mainly prepared through interfacial reaction and simultaneous dynamic vulcanization [26,27,28,29,30,31,32,33,34]. Natural rubber (NR) was graft-modified to prepared NR-graft-glycidyl methacrylate (GNR), and PLA/GNR thermoplastic vulcanizates (TPVs) with excellent toughness were obtained by in situ dynamical vulcanizing [30]. However, only a few studies have focused on the improvement of the flame retardancy of super-tough PLA TPVs. Therefore, it is necessary to simultaneously improve the flame retardancy and toughness of PLA to broaden the potential application of degradable PLA materials. 

In this study, a modified aluminum hypophosphite by silane (SiAHP) was prepared to improve the compatibility between the flame retardant and polymer matrix. The SiAHP was incorporated into the PLA/GNR thermoplastic vulcanizates to fabricate a series of toughened and flame-retardant PLA-based composites. The mechanical properties, morphology, and flame retardancy of flame retardant (FR) PLA/GNR and PLA/GNR/SiAHP composites were systematically characterized. The thermal properties and flame-retardant mechanism of PLA/GNR/SiAHP composites were investigated.

## 2. Materials and Methods 

### 2.1. Materials 

PLA pellets (2003D, $\bar{M_{W}}$: 38 × 104 g mol^−1^, D = 1.59, ρ = 1.26 g cm^−3^, melt mass flow rate (MFR) (6 g/10 min, 210 °C, 2.16 kg) were procured from Natureworks Company (US). Modified natural rubber grafted by glycidyl methacrylate (GNR) was prepared in our lab according to a previous paper [34]. Dicumyl peroxide (DCP) was produced by Hunan Yixiang Technology Co., Ltd. (Liuyang, China). Antioxidant 1010 was purchased from Shandong Linyi Sanfeng Chemical Co., Ltd. (Linyi, China). The AHP was provided by Qingdao Fusilin Chemical and Technology Corp. (Qingdao, China).

### 2.2. Preparation of Flame-Retardant PLA/GNR/SiAHP Composites

The AHP was first modified by N-(β-aminoethyl)-γ-aminopropylmethyldimethoxy silane (APTS) to obtain SiAHP, according to the previous literature [35]. The PLA, 20 wt.% GNR, and 0.2 wt.% antioxidant 1010 were mixed in a Haake chamber at 135 °C and at a rotor speed of 60 rpm for 5 min, and DCP (1.5 wt.% of GNR) was added to initiate the dynamic vulcanization of GNR for 5 min, and AHP or SiAHP (16, 18, 20 wt.%) was added to blend with PLA thermoplastic vulcanizate(TPV) for 5 min to prepare the PLA/GNR/AHP and PLA/GNR/SiAHP composites. The flame-retardant PLA/GNR composite was pressed into a sheet at 180 °C for 5 min under 10 MPa. The samples were prepared by cutting standard sizes to measure the flame retardancy and mechanical properties. 

### 2.3. Characterization

Stress–strain curves of samples were measured using a cross-head rate of 50 mm/min according to the ASTM D882 standard on a screw-driven universal testing machine (AL-7000M, Taiwan Gotech Testing Machines Inc. Taichung, Taiwan). 

The Izod notched impact strengths were tested according to ISO 180 using an impact tester (Suzhou Ligao Detection Equipment Co., Ltd., Suzhou, China). 

Surface morphologies of the char residue were measured using scanning electron microscopy (SEM) (JSM-6700F, Japan Electronics Corp. Tokyo, Japan). The surface of samples was uniformly sputter-coated with a thin layer of gold prior to examination.

The thermogravimetric (TG) curve was measured on a Perkin–Elmer TGA-7 apparatus at a heating rate of 10 °C/min from 30 to 700 °C under a 40 mL/min flowing N_2_.

The LOI values of samples were obtained from a JF-3 oxygen index apparatus (Jiangning, China) according to ASTM D2863-97, and the size of the samples was 130 × 6.5 × 3.2 mm^3^.

The UL-94 vertical burning test was conducted by a CFZ-1-type apparatus (Jiangning, China) according to ASTM UL 3801–2010, and the size of specimens was 130 × 13 × 3.2 mm^3^. 

The cone calorimeter test was carried out using a cone calorimeter (Suzhou Vouch Testing Technology Co., Ltd., type, 6810, Suzhou, China) under a heat flux of 50 kW/m^2^ with a sample size of 100 × 100 × 4 mm^3^ according to the ASTM E1354 standard.

## 3. Results

### 3.1. Mechanical Properties and Morphology

**Figure 1:**
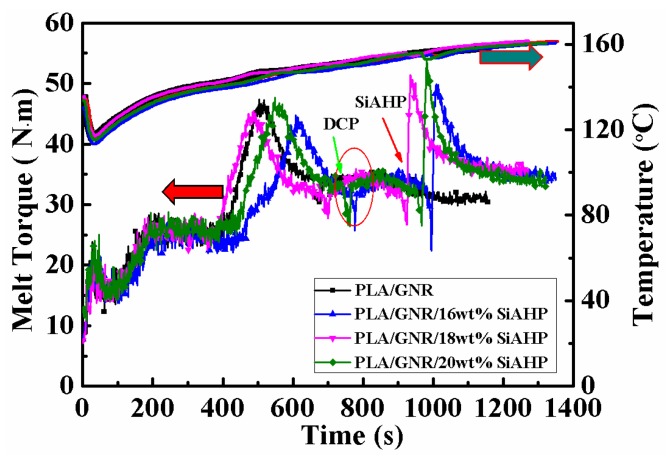
Melt torque–time curves of the PLA/GNR/SiAHP composites.

The dynamical vulcanizing process of the PLA/GNR and melt-blending evolution of the PLA/GNR/SiAHP composites are shown in Figure 1. With the incorporation of 20 wt.% GNR, the melt torque of PLA/GNR increased and reached its first peak value of melt torque (44.8 N.m). After the addition of 1.5 wt.% DCP, the melt torque slightly increased due to the occurrence of dynamical vulcanization of GNR. After adding 16 wt.% SiAHP, the melt torque greatly increased and reached a higher peak of melt torque of 49.9 N.m at 1009 s, and then the melt torque gradually decreased with time. When the content of SiAHP increased to 18 wt.% and 20 wt.%, the peak values of melt torque of PLA/GNR composites were slightly increased at the second melt-blending stage due to the increase of the dynamic viscosity of PLA/GNR composites.

**Figure 2:**
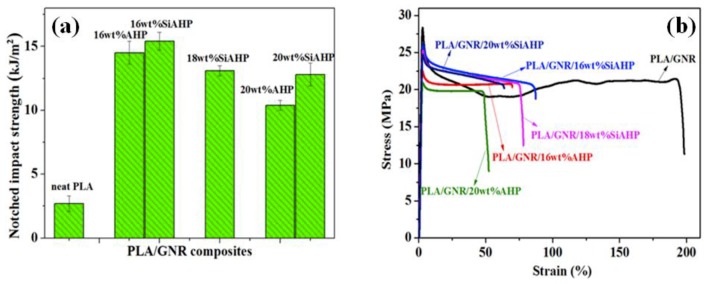
Mechanical properties of PLA, PLA/GNR TPV, PLA/GNR/AHP, and PLA/GNR/SiAHP composites: (**a**) notched impact strength, (**b**) stress–strain curves.

Table 1 shows the mechanical properties of the PLA/GNR TPVs with different contents of flame retardants. Figure 2a,b shows the impact strength and stress–stain curves of the PLA/GNR/AHP and PLA/GNR/SiAHP composites, respectively. The notched impact strength of the PLA/20wt%GNR TPV dramatically improved from 2.7 kJ/m^2^ of neat PLA to 65.0 kJ/m^2^, and the elongation at break increased from 4% of neat PLA to 183%. With the incorporation of 16 wt.%AHP, the notched impact strength and elongation of the PLA/GNR/16wt%AHP composites decreased to 14.5 kJ/m^2^ and 68%. The decrease in the toughness of PLA/GNR/AHP was mainly ascribed to the fact that the AHP was a kind of inorganic phosphorus-containing flame retardant. After the modification of AHP, the notched strength and elongation of the PLA/GNR/16wt%SiAHP composite were slightly higher than those of the PLA/GNR/16wt%AHP composite, respectively. It was because the surface modification of SiAHP could improve the interfacial interaction between PLA/GNR matrix and flame retardant to a certain extent. When the SiAHP increased to 18 wt.%, the notched strength and elongation of the PLA/GNR/18wt%SiAHP composite were 13.1 kJ/m^2^ and 72%, which were 385% and 17 times higher than those of neat PLA, respectively. The results demonstrated the PLA/GNR/SiAHP composites still exhibited good toughness.

**Figure 3:**
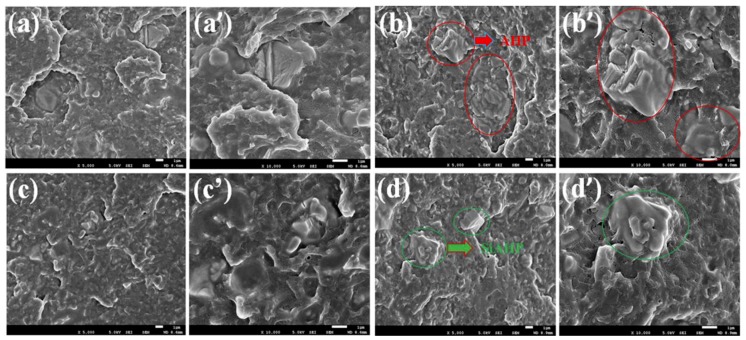
SEM images of cryo-fractured surface for PLA/GNR/AHP and PLA/GNR/SiAHP composites. (**a**, **a’**) PLA/GNR/16wt%AHP; (**b**, **b’**) PLA/GNR/20wt%AHP; (**c**, **c’**) PLA/GNR/16wt% SiAHP; (**d**, **d’**) PLA/GNR/20wt%SiAHP.

The dispersion of AHP and SiAHP in the flame-retardant PLA/GNR composites can be observed in Figure 3. With the incorporation of 16 wt.% AHP, some irregular AHP particles were distributed in the PLA/GNR composite, and the dimensions of these particles were in the range of 2.0–5.0 µm (Figure 3a,a’). When the content of AHP was increased to 20 wt.%, there was obvious aggregation of AHP particles in the PLA/GNR matrix (Figure 3b,b’). In the case of PLA/GNR/16wt%SiAHP composites, the dispersion of SiAHP particles became more uniform (Figure 3c,c’). With the increasing SiAHP content, most of the SiAHP particles did not aggregate in the PLA/GNR/20wt%SiAHP composite (Figure 3d,d’).

**Figure 4:**
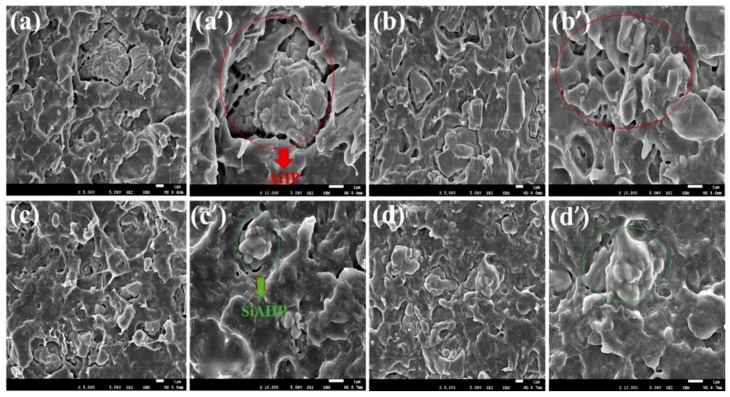
SEM images of the impact-fractured surface for the PLA/GNR/AHP and PLA/GNR/SiAHP composites. (**a**, **a’**) PLA/GNR/16wt%AHP; (**b**, **b’**) PLA/GNR/20wt%AHP; (**c**, **c’**) PLA/GNR/16wt% SiAHP; and (**d**, **d’**) PLA/GNR /20wt%SiAHP.

The surface phase morphologies of the impact-fractures of the PLA/GNR/AHP and PLA/GNR/SiAHP composites were compared in Figure 4. For the PLA/GNR/16wt%AHP and PLA/GNR/20wt%AHP composites, it was observed that most of the AHP particles were de-bonded from the matrix under impact energy (Figure 4a,b), because the interfacial interaction between the PLA/GNR matrix and AHP particles was poor (Figure 4a’,b’). For the PLA/GNR/16wt%SiAHP and PLA/GNR/20wt%SiAHP composites, these irregular SiAHP particles were evenly distributed in the PLA/GNR composites (Figure 4c,d), and some SiAHP particles were adhered at the PLA/GNR matrix under impact strength (Figure 4c’,d’). The results confirmed that the modification of SiAHP could promote the interfacial compatibility between the flame retardant and the PLA/GNR matrix. This was mainly because the chemical interfacial reaction between the amino groups of the silane molecule in the modified SiAHP and the epoxy groups in the GNR macromolecular chains promoted the compatibility between the flame retardant and PLA/GNR matrix.

### 3.2. Thermal Stability

**Figure 5:**
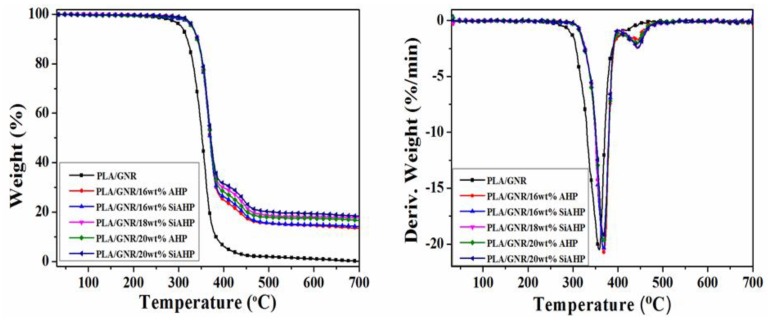
TG and DTG curves of the PLA/GNR and FR PLA/GNR composites under N_2_ atmosphere.

The TG data of the PLA/GNR and flame-retardant PLA/GNR composites are listed in Table 2. For the PLA/GNR/16wt%AHP composite, the onset decomposition temperature (*T_5%_*) was increased from 306 °C of the PLA/GNR to 329.0 °C, and the two maximum mass loss temperatures (*T_max_*_1_ and *T_max_*_2_) were increased to 366.4 and 443.3 °C, which corresponded to the thermal decomposition of the PLA/GNR matrix and AHP, respectively, and the char yield of the PLA/GNR/16wt%AHP composite at 700 °C was increased from only 0.2% of the PLA/GNR TPV to 13.4%. Figure 5 shows the TG and differential thermogravimetric (DTG) curves of the PLA/GNR TPV and PLA/GNR composites under N_2_ atmosphere. By the surface modification, the char yield of the PLA/GNR/16wt%SiAHP composite was increased to 17.6%, which was obviously higher than that of PLA/GNR/16wt%AHP composite. With an increase of SiAHP content, the char yield of the PLA/GNR/20wt%SiAHP composite increased to 24.0%. It indicated that the surface modification of SiAHP obviously enhanced the charring ability of the PLA/GNR/SiAHP composites. 

### 3.3. Flame Retardancy of PLA/GNR Composites

Table 3 shows LOI and UL-94 test results of the PLA/GNR and FR PLA/GNR composites. The LOI value of the PLA/GNR TPV was only 19.0% and its UL-94 testing was no rating, meanwhile it produced a large amount of melt-dripping during the combustion. With the incorporation of 16 wt.% AHP, the LOI value of PLA/GNR/AHP composite raised it up to 25.5% and its UL-94 test was no rating. In the case of the PLA/GNR/16wt%SiAHP composite, it passed the UL-94 V-1 rating. With the addition of 18 wt.% and 20 wt.% SiAHP, the LOI values of the PLA/GNR/SiAHP composites increased to 26.5% and 27.0%, respectively, and the UL-94 tests passed the V-0 rating. The results indicated that the flame retardancy of PLA/GNR/SiAHP was enhanced by the surface modification.

**Figure 6:**
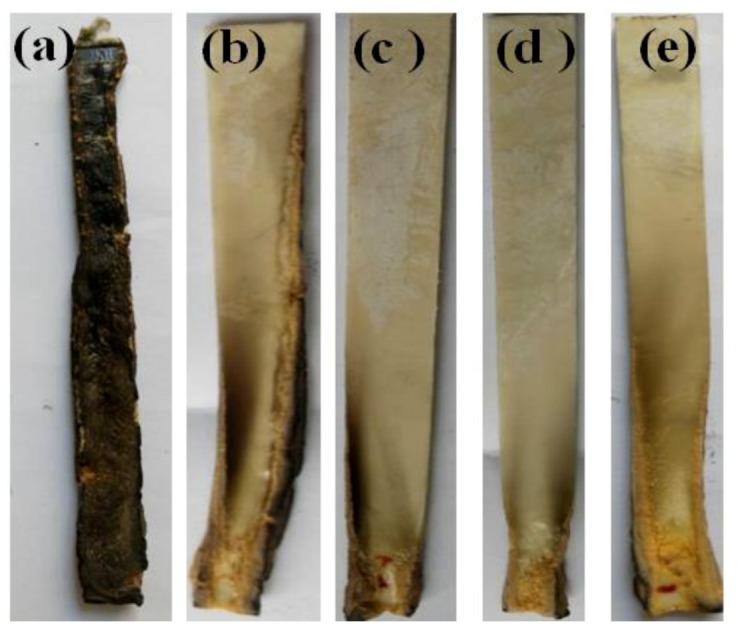
Digital photographs of PLA/GNR and PLA/GNR/SiAHP composites after UL-94 vertical burning tests. (**a**) PLA/GNR/16wt%AHP; (**b**) PLA/GNR/16wt%SiAHP; (**c**) PLA/GNR/18wt%SiAHP; (**d**) PLA/GNR/20wt%AHP; (**e**) PLA/GNR/20wt%SiAHP.

In the UL-94 vertical burning test, the PLA/GNR TPV rapidly burned and continuously produced severe melt-dripping, and then no residue was left. Figure 6a–e shows the digital photos of the residues of the PLA/GNR/AHP and PLA/GNR/SiAHP composites. For the PLA/GNR/16wt%AHP composite, it continued to burn and produced a large amount of smoke. In the case of the PLA/GNR/16wt%SiAHP composite, it burned for less time and produced less smoke, and the melt dripping of the PLA was effectively suppressed (Figure 6b). When the SiAHP content exceeded 18 wt.% or the AHP content exceeded 20 wt.%, the burning of the PLA/GNR composites rapidly extinguished, and nearly no smoke and melt drippings were generated, and then only a small amount of char residue was formed at the surface of the samples (Figure 6c–e). 

**Figure 7:**
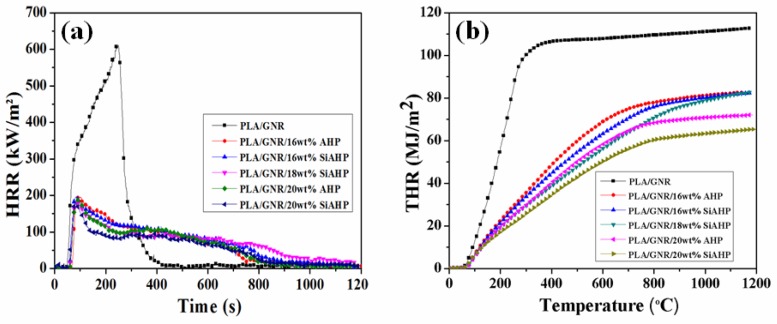
Heat release rate (HRR) (**a**) and total release rate (THR) (**b**) curves of the PLA/GNR TPV and FR PLA/GNR composites.

The cone calorimeter test was conducted to study the flame-retardant mechanism of the PLA/GNR/AHP and PLA/GNR/SiAHP composites. The corresponding cone calorimeter test data are summarized in Table 4. Figure 7 shows the heat release rate (HRR) and total heat release (THR) curves of the PLA/GNR TPV and flame-retardant PLA/GNR composites. The PLA/GNR TPV presented a sharp peak of heat release rate value (pHRR) of 612.2 kW/m^2^ at 47 s (Figure 7a). With the addition of 16 wt.% AHP, the pHRR value was dramatically decreased to 198.0 kW/m^2^, and the TTI was extended to 58 s. With an increase of AHP, the pHRR value of PLA/GNR/20wt%AHP composite was decreased. In the case of the PLA/GNR/20wt%SiAHP composite, the pHRR value was decreased to 178.0 kW/m^2^, which was lower than that of the PLA/GNR/20wt%AHP composite. As shown in Figure 7b, the THR value of the PLA/GNR/16wt%SiAHP composite was reduced from 112.7 MJ/m^2^ of the PLA/GNR TPV to 82.4 MJ/m^2^. The THR value of the PLA/GNR/20wt%SiAHP composite decreased further to 70.2 MJ/m^2^, which was lower than that of the PLA/GNR/20wt%AHP composite. These results demonstrated that the flame retardancy of the PLA/GNR/SiAHP composites were more effectively decreased by the surface modification.

**Figure 8:**
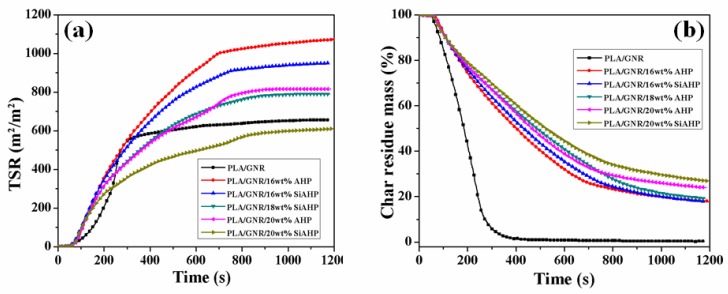
Total smoke release (TSR) (**a**) and char residue mass (CR) (**b**) curves of PLA/GNR TPV and FR PLA/GNR composites.

The total smoke release (TSR) and char residue (CR) yield curves of the PLA/GNR composites with different contents of AHP and SiAHP are shown in Figure 8a,b, respectively. The TSR values of the PLA/GNR/16wt%AHP and PLA/GNR/16wt%SiAHP composites were dramatically higher than that of PLA/GNR TPV. This was because AHP and SiAHP released phosphorus-containing compounds in the gaseous phase to trap radicals (e.g., H, HO radicals) and interrupt the decomposition of the PLA macromolecular chain [35,36]. In addition, the SiAHP (AHP modified by APTS) easily decomposed into inert gases (such NH_3_) to dilute the concentration of combustible gases. It was suggested that AHP or SiAHP in PLA/GNR composites presents gaseous-phase flame retardant mechanisms [35,36,37]. As shown in Figure 8b, the char residue yield of PLA/GNR/AHP and PLA/GNR/SiAHP composites increased with the increase in the content of AHP or SiAHP. The more char residues of PLA/GNR composites can effectively isolate the release of combustible gases into the interior polymer and heat transfer, PLA/GNR/AHP, and PLA/GNR/SiAHP composites also presented a condensed-phase flame retardant mechanism. Thus, AHP or SiAHP in PLA/GNR composites played a role in both condensed-phase and gaseous-phase flame retardancy. 

As shown in Table 4, for the PLA/GNR/20wt%SiAHP composite, the TSR value decreased to 609.1 m^2^·m^−2^, which was lower than that of the PLA/GNR/20wt%AHP composite, and the char yield (CR) of the PLA/GNR/20wt%SiAHP composite increased to 26.9%, which was obviously higher than that of the PLA/GNR/20wt%AHP composite (24.0%). Thus, the more char residue from the PLA/GNR/20wt%SiAHP composite kept from the release of combustible gases resulted in a further decrease in the TSR value. The results indicated that PLA/GNR/SiAHP composites exhibited a slightly higher charring ability and smoke suppression compared to the corresponding PLA/GNR/AHP composites.

**Figure 9:**
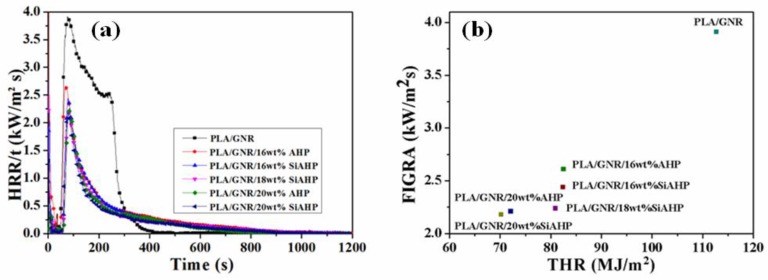
HRR(t)/t versus time curves of PLA/GNR TPV and FR PLA/GNR composites (**a**) and FIGRA versus THR (**b**).

The maximum ratio HRR(t)/t) and pHRR/t_ign_ in the cone calorimeter test represent the fire growth rate (FIGRA) and the flame spread rate, respectively. Figure 9a,b shows the HRR(t)/t curves and the corresponding FIGRA of the PLA/GNR TPV and FR PLA/GNR composites. The FIGRA and pHRR/t_ign_ values of PLA/GNR TPV and PLA/GNR composites are listed in Table 5. The pHRR/t_ign_ values decreased with an increase in the content of AHP and SiAHP, and the FIGRA and pHRR/t_ign_ values of PLA/GNR/SiAHP composites were lower than those of the corresponding PLA/GNR/AHP composites, respectively. The lower FIGRA and pHRR/t_ign_ values demonstrate the better fire hazard safety of the materials. Thus, SiAHP is a more effective flame retardant in enhancing the fire hazard safety of the PLA/GNR TPVs. 

### 3.4. Char Morphology 

**Figure 10:**
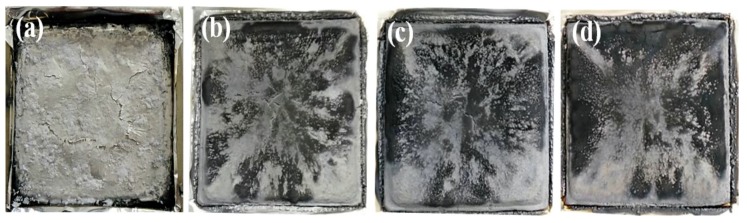
Digital photos of char residues for PLA/GNR TPV (**a**); PLA/GNR/16wt%SiAHP (**b**); PLA/GNR/18wt%SiAHP (**c**); PLA/GNR/20wt%SiAHP (**d**) after the cone calorimeter test.

Figure 10 shows the digital photographs of the burning residues of the PLA/GNR TPV and PLA/GNR/SiAHP composites after the cone calorimeter test. For PLA/GNR TPV, a thin layer of gray carbonized residue was formed (Figure 10a). For PLA/GNR/16wt%SiAHP composite, the residue exhibited a thick char structure with a few small cracks, and small amount of black graphite was distributed at the surface of residue (Figure 10b). With an increase of SiAHP content, the residue became more compact (Figure 10c,d). In the case of the PLA/GNR/20wt%SiAHP composites, there were almost no obvious cracks at the surface of the residue. It reveals that the incorporation of SiAHP promoted the formation of a compact char structure of PLA/GNR composites. This kind of the compact structure of residue could effectively insulate the diffusion of flammable gases into the interior of polymer. 

**Figure 11:**
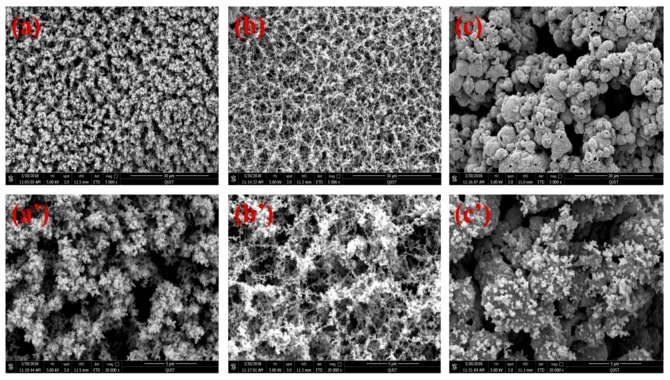
SEM images of char residues for PLA/GNR/SiAHP composites. (**a**, **a’**) PLA/GNR/16wt% SiAHP; (**b**, **b’**) PLA/GNR/18wt%SiAHP; (**c**, **c’**) PLA/GNR/20wt%SiAHP.

The surface microstructure of the residues of the PLA/GNR/SiAHP composites were investigated by SEM observation. For PLA/GNR/16wt%SiAHP, a kind of porous char layer surface structure was shown in Figure 11a. In the enlarged micrograph (Figure 11a’), it was observed that many nanoscale particles were aggregated at the surface of char layer. In the case of the PLA/GNR/18wt%SiAHP, the residue displayed more even and smaller porous structure (Figure 11b). In the enlarged micrograph (Figure 11b’), many nanoscale particles were connected to form a three-dimensional network structure at the surface of residue. In case of the PLA/GNR/20wt%SiAHP composite (Figure 11c), it was observed that many different sizes of particles were distributed in the residue. In the enlarged magnification, it was found that small nanoparticles were attached at the surface of microscale particles (Figure 11c’). These nano- and microscale particles were suggested as the thermal decomposition product of PLA/GNR matrix and SiAHP, respectively.

In the PLA/GNR/SiAHP composites, SiAHP generated phosphorus-containing gaseous substance (such as PH_3_), which could act to generate P and PO in the gaseous phase during the combustion to capture the highly reactive radicals (HO, H), and hence, extinguish the flame. Meanwhile, the compact char layer containing polyphosphate could effectively insulate the diffusion of combustible gases, and further protect the inner polymer from decomposing [35,36]. Therefore, the flame-retardant mechanism of the PLA/GNR/SiAHP composites is considered to be a synergistic effect of the gaseous flame retardant and condensed flame-retardant mechanisms. In particular, the melt-dripping behavior of the PLA matrix could be effectively suppressed by the protective barrier of char layer. 

## 4. Conclusions

In this study, a toughened and good flame-retardant PLA/GNR/SiAHP composite was prepared by sequentially dynamically vulcanizing and reactive melt-blending. The surface modification of AHP enhanced the interfacial compatibility between the SiAHP and PLA matrix and charring ability of PLA/GNR/SiAHP composites to a certain extent, the toughness and flame retardancy of the PLA/GNR/SiAHP composites were slightly higher than those of the corresponding PLA/GNR/AHP composites, respectively. The notched impact strength and elongation of the PLA/GNR/18wt%SiAHP composite were approximately 3.9 and 17 times higher than those of neat PLA, respectively. The LOI of the PLA/20wt%GNR TPV with 18 wt.% SiAHP increased to 26.5%, and its UL-94 test passed V-0 rating. Importantly, the addition of SiAHP completely inhibited the melt-dripping behavior of the PLA/GNR/SiAHP composite. The pHRR and THR values of PLA/GNR/SiAHP composite were dramatically decreased. The good flame retardancy of SiAHP in PLA/GNR composites was suggested to the synergistic effect of gaseous flame retardant and condensed phase flame retardant mechanisms. Moreover, the high char residue yield and compact protective char layer of PLA/GNR/SiAHP composite endowed it excellent melt-dripping resistance.

## Figures and Tables

**Table 1:** Mechanical properties of the PLA/NR and FR PLA/GNR composites.

Sample	Notched Impact Strength (kJ.m^−2^)	Tensile Strength (MPa)	Elongation at Break (%)
PLA	2.7 ± 0.6	68.9 ± 4.1	4 ± 0
PLA/GNR/16wt%AHP	14.5 ± 0.9	22.0 ± 1.9	68 ± 11
PLA/GNR/20wt%AHP	10.4 ± 0.4	20.5 ± 0.7	59 ± 8
PLA/GNR/16wt%SiAHP	15.4 ± 0.7	25.6 ± 1.0	78 ± 7
PLA/GNR/18wt%SiAHP	13.1 ± 0.4	24.5 ± 1.4	72 ± 13
PLA/GNR/20wt%SiAHP	12.8 ± 0.9	23.1 ± 1.4	64 ± 8
PLA/GNR	65.0 ± 7.8	28.8 ± 1.2	183 ± 20

**Table 2:** TG data of the PLA/GNR TPV and FR PLA/GNR composites under N_2_ atmosphere.

Sample	*T_5%_* (°C)	*T_max_*_1_ (°C)	*T_max_*_2_ (°C)	Char Yield at 700 °C (%)
PLA/GNR TPV	306.0	358.8	-	0.2
PLA/GNR/16wt%AHP	329.0	366.4	443.3	13.4
PLA/GNR/20wt%AHP	332.0	369.5	446.4	16.7
PLA/GNR/16wt%SiAHP	329.0	368.2	444.7	17.6
PLA/GNR/18wt%SiAHP	331.0	369.5	445.3	19.2
PLA/GNR/20wt%SiAHP	332.0	370.7	446.7	24.0

**Table 3:** LOI and UL-94 test results of the PLA/GNA TPV and FR PLA/GNR composites.

Sample	LOI (%)	UL-94 Rating	Smoke	Dripping
PLA/GNR TPV	19.0	NR	less	Y
PLA/GNR/16wt%AHP	25.5	NR	more	N
PLA/GNR/20wt%AHP	26.5	V-0	less	N
PLA/GNR/16wt%SiAHP	26.0	V-1	more	N
PLA/GNR/18wt%SiAHP	26.5	V-0	less	N
PLA/GNR/20wt%SiAHP	27.0	V-0	no	N

**Table 4:** Cone calorimeter data of the PLA/GNR and FR PLA/GNR composites.

Sample	TTI (s)	pHRR (kW·m^−2^)	T_pHRR_ (s)	THR (MJ·m^−2^)	TSR (m^2^·m^−2^)	CR (%)
PLA/GNR TPV	47 ± 2	612.2 ± 3.1	244 ± 2	112.7 ± 2.3	656.0 ± 5.3	0.2
PLA/GNR/16wt%AHP	58 ± 2	198.0 ± 1.7	83 ± 2	82.5 ± 1.6	1041.6 ± 3.4	17.6
PLA/GNR/20wt%AHP	60 ± 1	189.7 ± 2.1	86 ± 2	72.1 ± 1.3	805.8 ± 2.8	24.0
PLA/GNR/16wt%SiAHP	59 ± 2	196.9 ± 2.5	81 ± 1	82.4 ± 1.5	952.7 ± 3.7	18.0
PLA/GNR/18wt%SiAHP	61 ± 1	185.8 ± 1.6	88 ± 2	80.9 ± 2.0	789.9 ± 1.9	19.3
PLA/GNR/20wt%SiAHP	62 ± 1	178.0 ± 2.2	82 ± 1	70.2 ± 1.1	609.1 ± 2.8	26.9

**Table 5:** Cone calorimeter data of PLA/GNR TPV and FR PLA/GNR composites.

Sample	pHRR/t_ign_ (kW/(m^2^/s))	FIGRA (kW/(m^2^/s))
PLA/GNR TPV	13.03	3.91
PLA/GNR/16wt%AHP	3.41	2.61
PLA/GNR/16wt%SiAHP	3.33	2.44
PLA/GNR/18wt%SiAHP	3.04	2.24
PLA/GNR/20wt%AHP	3.16	2.21
PLA/GNR/20wt%SiAHP	2.87	2.18
